# Ontogeny influences sensitivity to climate change stressors in an endangered fish

**DOI:** 10.1093/conphys/cou008

**Published:** 2014-03-10

**Authors:** L. M. Komoroske, R. E. Connon, J. Lindberg, B. S. Cheng, G. Castillo, M. Hasenbein, N. A. Fangue

**Affiliations:** 1 Department of Wildlife, Fish & Conservation Biology, University of California, Davis, Davis, CA 95616, USA; 2 Department of Anatomy, Physiology and Cell Biology, School of Veterinary Medicine, University of California, Davis, Davis, CA 95616, USA; 3 Fish Conservation and Culture Facility, University of California, Davis, Davis, CA 95616, USA; 4 Department of Environmental Science and Policy, University of California, Davis, Davis, CA 95616, USA; 5 United States Fish and Wildlife Service, Lodi, CA 95240, USA; 6 Aquatic Systems Biology Unit, Department of Ecology and Ecosystem Management, Technische Universität München, Muehlenweg 22, D-85354 Freising, Germany

**Keywords:** Conservation management, estuarine fishes, salinity tolerance, thermal tolerance

## Abstract

Coastal ecosystems are among the most human-impacted habitats globally, and their management is often critically linked to recovery of declining native species. In the San Francisco Estuary, the Delta Smelt (*Hypomesus transpacificus*) is an endemic, endangered fish strongly tied to Californian conservation planning. The complex life history of Delta Smelt combined with dynamic seasonal and spatial abiotic conditions result in dissimilar environments experienced among ontogenetic stages, which may yield stage-specific susceptibility to abiotic stressors. Climate change is forecasted to increase San Francisco Estuary water temperature and salinity; therefore, understanding the influences of ontogeny and phenotypic plasticity on tolerance to these critical environmental parameters is particularly important for Delta Smelt and other San Francisco Estuary fishes. We assessed thermal and salinity limits in several ontogenetic stages and acclimation states of Delta Smelt, and paired these data with environmental data to evaluate sensitivity to climate-change stressors. Thermal tolerance decreased among successive stages, with larval fish exhibiting the highest tolerance and post-spawning adults having the lowest. Delta Smelt had limited capacity to increase tolerance through thermal acclimation, and comparisons with field temperature data revealed that juvenile tolerance limits are the closest to current environmental conditions, which may make this stage especially susceptible to future climate warming. Maximal water temperatures observed *in situ* exceeded tolerance limits of juveniles and adults. Although these temperature events are currently rare, if they increase in frequency as predicted, it could result in habitat loss at these locations despite other favourable conditions for Delta Smelt. In contrast, Delta Smelt tolerated salinities spanning the range of expected environmental conditions for each ontogenetic stage, but salinity did impact survival in juvenile and adult stages in exposures over acute time scales. Our results underscore the importance of considering ontogeny and phenotypic plasticity in assessing the impacts of climate change, particularly for species adapted to spatially and temporally heterogeneous environments.

## Abstract (precis)

We assessed thermal and salinity limits in several ontogenetic stages and acclimation states of Delta Smelt to evaluate sensitivity to climate change stressors. Thermal tolerance decreased among successive stages, and juvenile tolerance limits were closest to current environmental conditions. Salinity impacted juvenile and adult survival in exposures over acute timescales.

## Introduction

Coastal ecosystems are among the most human-impacted habitats globally (Lotze *et al.*, 2006), and climate change is predicted to interact with existing stressors to generate effects spanning multiple physical and biological scales (Harley *et al.*, 2006; Crain *et al.*, 2008). The San Francisco Estuary (SFE) is one of the largest and most economically valuable estuarine systems in North America (Service, 2007), and anthropogenic use of the SFE has resulted in it being one of the most modified and controlled systems in the world (Nichols *et al.*, 1986). Landscape-scale modifications have reduced habitat complexity and led to major declines of once numerous native species (Sommer *et al.*, 2007; Moyle *et al.*, 2010), exemplified by precipitous declines of multiple pelagic fish populations since the early 2000s (referred to as the pelagic organism decline; Feyrer *et al.*, 2007; Sommer *et al.*, 2007). One of the species affected by the pelagic organism decline is the Delta Smelt (*Hypomesus transpacificus*), a fish endemic to the SFE (Bennett, 2005).

Like many other species inhabiting human-altered ecosystems, the decline of Delta Smelt has been associated with numerous stressors, such as habitat loss, entrainment at water pumping stations (i.e. fish drawn through intakes; Castillo *et al.*, 2012), competition and predation from non-native species, food limitation due to changes in the plankton community, altered abiotic conditions and contaminants (Sommer *et al.*, 2007; Winder and Jassby, 2011; Brooks *et al.*, 2012; Cloern and Jassby, 2012). Listed under both the Federal Endangered Species Act and California Endangered Species Act and an indicator of SFE ecological health, recovery of Delta Smelt is a critical component of statewide management efforts to balance ecosystem restoration with ecosystem services. Specifically, resource managers are tasked with providing a reliable water supply for farmlands and over 23 million Californians, as well as the maintenance of healthy wildlife populations and ecosystem function (NRC, 2012). Determining the best management actions to accomplish these sometimes conflicting objectives has been politically and publically debated in California for over a decade, making the scientific understanding of stressor impacts on Delta Smelt important not only to effective conservation, but also to statewide environmental policies.

The SFE is subject to strong tidal influences from the Pacific Ocean mixing with fluctuating freshwater input from the Sacramento and San Joaquin Rivers (Cloern and Jassby, 2012), producing a dynamic system with spatial and seasonal gradients in water parameters (e.g. temperature, salinity). These variations in abiotic conditions may influence behavioural responses and affect physiological processes across multiple levels of biological organization in fishes such as Delta Smelt (Fry, 1971; Hochachka and Somero, 2002). Temperature is a key determinant of fish survival and performance (Brett, 1971), resulting in fishes being generally adapted to the water temperatures that they routinely experience (Fangue *et al.*, 2006; Eliason *et al.*, 2011). Likewise, salinity is a critical abiotic condition for fishes, and tolerance is largely dependent on physiological responses to maintain ionic and osmotic balance (reviewed by Evans, 2008). Coping with temperature and salinity stress can be associated with a high energetic cost, such that optimal performance often occurs over a relatively narrow range for each parameter (Schulte *et al.*, 2011; Hasenbein *et al.*, 2013). Yet temperature and salinity tolerance limits are governed by the complex interplay of mechanisms of adaptation and phenotypic plasticity, such as acclimatization, i.e. reversible biochemical changes due to environmental exposure that can alter tolerance within individuals (Schulte *et al.*, 2011). Individuals may be able to employ phenotypic plasticity to cope with thermal or salinity stress within a range of conditions; however, beyond a certain threshold, evolutionary adaptation via natural selection is necessary to avoid extirpation or, in the case of endemic fishes such as Delta Smelt with extremely limited options for range expansion, extinction (Hofmann and Todgham, 2009, McBryan *et al.*, 2013).

**Figure 1:**
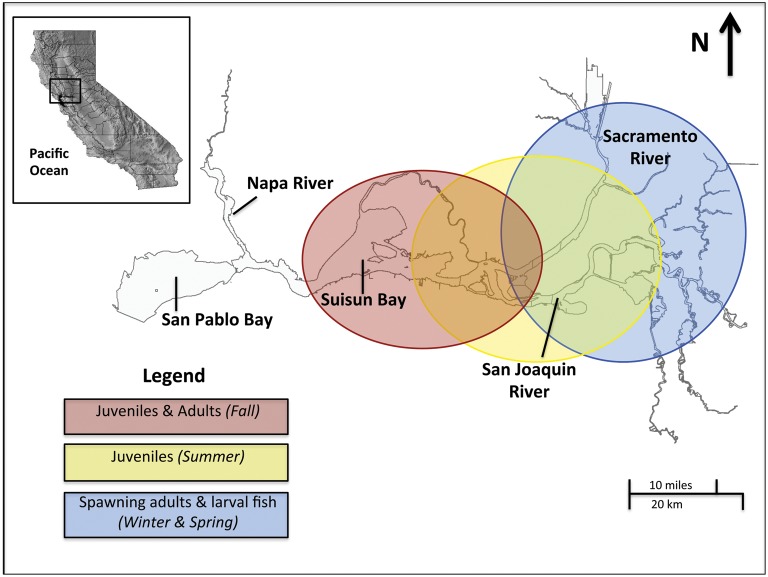
Map of the San Francisco Estuary depicting estimated habitat regions for different ontogenetic stages of Delta Smelt, where abiotic conditions, including salinity and temperature, fluctuate spatially and temporally.

The life history of Delta Smelt is composed of a largely annual life-cycle, in which life stages vary both spatially and seasonally in the SFE. Adults inhabit the lower SFE and migrate upstream annually in the late autumn to early winter to spawn (Fig. 1), and only a small percentage of adults survive their first spawn (Bennett, 2005). Larval fish develop in freshwater habitats until migrating downstream as juveniles towards the low-salinity zone (1–6 ppt) in late spring, where they remain throughout the summer and early autumn as they mature into adults (Bennett, 2005). Although Delta Smelt are not extremely strong swimmers (Swanson *et al.*, 1998), they are thought to use tidal currents to accomplish this migrational pattern (Moyle *et al.*, 2010). Water temperatures in the SFE usually peak in late summer and are lowest during winter months, with the highest temperatures occurring in freshwater habitat upstream (e.g. range 10–29°C for 2002–10; Fig. 1; Kimmerer, 2004; CDFW, 2013). Salinity increases from freshwater in upstream headwaters to seawater (∼34 ppt) commonly westward of Suisun Bay; however, the geographical position of the salinity gradient can also fluctuate temporally on scales from daily to seasonal to annual (CDFW, 2013). Thus, the dynamic environmental conditions combined with the seasonal migrations and the primarily annual life-cycle of Delta Smelt present distinct environmental conditions to each ontogenetic stage (Bennett, 2005; Moyle *et al.*, 2010). This may confer differential tolerance for temperature and salinity among ontogenetic stages and result in particular stages having higher sensitivity to environmental change.

Understanding the influences of ontogeny and acclimatization responses on the temperature and salinity tolerance of the Delta Smelt is particularly important because forecasted impacts of climate change in SFE include increases in the mean and variability of these environmental parameters (Cayan *et al.*, 2008; Cloern *et al.*, 2011). These climatic effects will be likely to augment the impacts of anthropogenic water diversion, which has already reduced the amount of freshwater inflow into the SFE by ∼40% on average (Lund *et al.*, 2010), resulting in higher salinities upstream during the autumn (Cloern and Jassby, 2012) and increased summer water temperatures (Moyle *et al.*, 2010). As these changes occur, the ability of Delta Smelt and other SFE fishes to maintain fitness in their native habitats will depend on their tolerance limits, phenotypic plasticity, adaptive capacity and related biotic interactions (Helmuth, 2009; Hofmann and Todgham, 2009). Furthermore, as a largely annual species, Delta Smelt recruitment is almost exclusively dependent on the fitness of the cohort in the previous year (Bennett, 2005); combined with their limited range, this may make Delta Smelt susceptible to declines due to poor environmental conditions in a single year.

As management plans are designed and implemented in the SFE to protect and restore wildlife in the face of climate change (e.g. on-going planning of large-scale tidal marsh restoration by the US Fish and Wildlife Service), understanding the physiological responses of organisms is paramount to effective conservation (Wikelski and Cooke, 2006). Previous studies of the thermal and salinity tolerance of Delta Smelt reported physiological limits for adults at one acclimation temperature (Swanson *et al.*, 2000), which are currently used for climate-change impact assessments (Cloern *et al.*, 2011, Brown *et al.* 2013) across the entire SFE and specific management regulations, such as restriction of water diversion flows to protect larval and juvenile Delta Smelt (USFWS, 2008). In this study, we assess thermal and salinity tolerance across ontogenetic stages and acclimation conditions to investigate the phenotypic plasticity in Delta Smelt responses to these climate-change stressors. We then employ several tolerance indices to evaluate the ‘buffer’ between Delta Smelt physiological limits and current habitat conditions to demonstrate differential sensitivity to climate warming among ontogenetic stages in this endangered species. Our study underscores the importance of assessing differences across life-cycles to climate change projections, particularly for species adapted to spatially and temporally heterogeneous environments.

## Materials and methods

### Fish culture and holding conditions

Fish were spawned between February and April in 2012 or 2013 and reared in optimal culture conditions (15.4–16.7°C) determined for Delta Smelt at the UC Davis Fish Conservation and Culture Laboratory (FCCL; Byron, CA, USA; Baskerville-Bridges *et al.*, 2005; Lindberg *et al.*, 2013). The Delta Smelt refuge population breeding programme at FCCL incorporates a unique genetic management strategy that includes a variety of methods to minimize inbreeding, maintain genetic representation from the wild founding population and maximize genetic diversity (Fisch *et al.*, 2009, 2013). We conducted experiments for five ontogenetic stages defined by days post hatch (dph), as follows: larval (30–32 dph), late-larval (60–64 dph), juvenile (140–164 dph), adult (200–250 dph) and post-spawning adults (>300 dph). We performed larval and late-larval fish experiments at FCCL, where fish were held under a natural photoperiod and fed live prey from cultures of rotifers (*Brachionus plicatus*) and brine shrimp nauplii (*Artemia franciscana*); *Nannochloropsis* (Reed Mariculture, Campbell, CA, USA) was used to increase water turbidity to promote feeding (Baskerville-Bridges *et al.*, 2004). We conducted experiments for post-larval stages (juveniles, adults and post-spawning adults) at the UC Davis Putah Creek aquaculture facility. Post-larval fish were fed an *ad libitum* 2:1 mixture of Inve-NRD commercial feed (Inve Aquaculture, Salt Lake City, UT, USA) and Hikari plankton (Pentair Aquatic Ecosystems, Apopka, FL, USA) throughout the day via automatic feeders under a natural photoperiod. Water quality was monitored daily with a YSI 556 water quality instrument (YSI Incorporated, Yellow Springs, OH, USA) for pH (8.6 ± 0.38) and dissolved oxygen (100–105% saturation). We used biological filtration, via a custom wet–dry filter that trickled water over Bio-Balls in an oxygen-rich chamber, with flushing to remove nitrogenous waste, and monitored ammonia and nitrite daily using a colorimetric test kit (API, Calfont, PA, USA). All handling, care and experimental procedures used were reviewed and approved by the UC Davis Institutional Animal Care and Use Committee (IACUC Protocol # 16591).

### Chronic lethal thermal maximum experiments

We quantified upper thermal acclimation limits using chronic thermal tolerance methodology (Bennett *et al.*, 1997). We conducted chronic lethal thermal maximum (CLT_max_) experiments only for post-larval stages (i.e. juveniles, adults and post-spawning adults) because larval fish require low light, black containers, minimal disturbance and elevated turbidities for feeding, and these rearing conditions inhibit accurate mortality estimations (Baskerville-Bridges *et al.*, 2005). For each stage, we defined CLT_max_ as the highest temperature at which 50% (CLT_max50_) and 95% (CLT_max95_) morbidity was observed (Fields *et al.*, 1987; Bennett and Beitinger, 1997). Fish remained in three 340 l holding tanks after 3 week acclimation periods (at 18.7 ± 0.2°C) while we increased temperature by 1°C/day until 100% mortality. We recorded temperature twice daily using a YSI 556 water quality instrument (YSI Incorporated) calibrated to a laboratory standard thermometer and hourly via iBCod temperature loggers (Alpha Mach, Inc., Ste-Julie, QC, Canada) submerged in each experimental tank.

### Critical thermal maximum experiments

We determined upper temperature tolerance in Delta Smelt using critical thermal methodology (CTM; Beitinger *et al.*, 2000), specifically quantifying critical thermal maximum (CT_max_), defined as the upper temperature at which fish lose the ability to escape conditions that will ultimately lead to death (Cox, 1974). Larval and late-larval fish only were held at optimal culture temperatures of 16.4 ± 0.25°C at FCCL, and we split post-larval fish held at the UC Davis Putah Creek facility into the following three acclimation groups: low (12.0–12.5°C); medium (15.5–16.5°C); and high (18.5–19.5°C; Table S1). We brought fish to each of the acclimation conditions by increasing or decreasing temperature by 1°C/day, and subsequently held fish at the final acclimation temperatures for at least 3 weeks prior to CT_max_ experiments (Beitinger *et al.*, 2000). For each CT_max_ trial, we placed a randomly selected fish in a 2 l black chamber filled with water at the respective acclimation temperature and covered with black mesh. We placed chambers in a 115 l water bath at acclimation temperature, and each chamber was fitted with an airstone to maintain dissolved oxygen at 100–105% saturation, a temperature logger and a glass thermometer calibrated to a laboratory standard thermometer. We fitted the water baths with titanium heaters, temperature controllers (Finnex Schuber Wright, Chicago, IL, USA) and Danner MD3 pumps (Pentair Aquatic Ecosystems, Apopka, FL, USA) to ensure even heating and circulation. Once in the chamber, each fish was given a 30–45 min habituation period prior to the start of the temperature increase. We used a thermal increase of 0.3°C/min for CT_max_ trials so that fish core temperatures would closely track changes in water temperature without allowing time for fish to acclimate thermally during the experiments (Becker and Genoway, 1979). We recorded temperatures and monitored fish for activity every 5 min until any abnormal behaviour was observed, after which we monitored fish continuously. We employed loss of equilibrium (LOE) as the end-point determining CT_max_, signifying ‘ecological death’ (Cox, 1974; Becker and Genoway, 1979; Beitinger *et al.*, 2000). Once LOE was reached, we recorded the temperature and immediately returned fish to adjacent chambers containing water at the fish's original acclimation temperature and allowed them to recover. Recovered fish were weighed (wet mass ± 0.1 g) and measured (fork length ± 0.5 mm) to assess covariation of fish size and treatments (Table S1), and returned to separate holding tanks to ensure they would not be selected for subsequent CT_max_ trials. We calculated CT_max_ as the arithmetic mean of the LOE temperatures for each stage and acclimation group (Cox, 1974; Beitinger *et al.*, 2000).

### Warming tolerance assessment

Warming tolerance (WT) is a measure of an organism's thermal buffer between the current habitat temperatures and its maximal thermal limits, with WT defined as the average amount of environmental temperature change an organism can tolerate before performance drops to fatal levels (Deutsch *et al.*, 2008). We calculated WT metrics for each ontogenetic stage as follows:

$$\hbox{WT} = \overline{\hbox{CT}_{\max}} - T_{{\rm habitat}}$$

where $\overline{\hbox{CT}_{\max}}$ is the mean CT_max_ determined for each ontogenetic stage for fish at medium acclimation temperature in experiments described above, and *T*_habitat_ is the metrics (median, 95th centile, 99th centile and maximum) of habitat water temperatures regionally and seasonally relevant for each ontogenetic stage during 2002–10. We used surface temperature data from SFE seasonal environmental monitoring surveys for *T*_habitat_, including the Fall Mid-Water Trawl (FMWT; September–December), 20 mm Survey (20 mm; April–June), Spring Kodiak Trawl (SKT; January–May) and Summer Townet Survey (TNS; June–August; CDFW, 2013). We constructed data sets of relevant habitat temperatures for each ontogenetic stage from survey data sets as follows: (i) standardizing duration by restricting all data sets to 2002–10; (ii) including only stations at which Delta Smelt were present during that time period; and (iii) including data seasonally relevant to each ontogenetic stage. Following steps (i)–(iii), the data used from selected stations in 2002–10 for each stage were as follows: larval, 20 mm; late-larval, 20 mm and TNS; juvenile, TNS and FMWT; adult, FMWT; and post-spawning adult, SKT. We then used constructed data sets to calculate *T*_habitat_ metrics and WT values for each stage.

### Chronic lethal salinity maximum experiments

We quantified upper salinity acclimation limits using chronic salinity tolerance methodology (Swanson *et al.*, 2000). Chronic lethal salinity maximum (CLS_max_) exposures were conducted for juvenile and adult Delta Smelt because chronic salinity exposure is environmentally relevant for these ontogenetic stages, and culture requirements for larval fish precluded their inclusion in CLS_max_ experiments (larval fish require turbidity for feeding that visually impedes accurate quantification of mortality over the time scales required for CLS_max_ experiments). Fish remained in three 340 l holding tanks after acclimation periods while we increased salinity by 2.0 ppt/12 h via the addition of artificial sea salt (Instant Ocean; Spectrum Brands, Inc., Blacksburg, VA, USA) into the sump of the recirculating system. We checked tanks for mortalities and recorded salinities every 12 h prior to the next salinity increase. We recorded salinities with a YSI 556 instrument, calibrated with 10 000 μS/cm National Institute of Standards and Technology traceable conductivity standard (YSI Incorporated). We increased salinity to 100% mortality or until seawater conditions were reached (34.0 ppt). If the latter conditions occurred, we held fish at 34.0 ppt for 3 weeks to monitor daily survival and assess salinity-related delayed mortality.

### Acute salinity maximum experiments

We determined upper salinity tolerance in Delta Smelt by conducting acute salinity maximum (AS_max_) exposures. The experimental design differed between larval and post-larval stages due to culture requirements and the environmental relevance of salinity end-points between stages. We used preliminary range-finding salinity experiments to determine treatment levels for juvenile and adult fish, and chose treatment levels for late-larval fish *a priori*, including three environmentally relevant salinities (0.4, 2.0 and 6.0 ppt) and two higher levels that late-larval fish may experience in rare conditions (12.0 and 18.0 ppt). For late-larval fish, we placed 15 individuals in each 9.5 l black container filled with holding tank water and fitted with airstones, mesh-covered drains and water lines to create flow-through conditions. Following an overnight acclimation period, we ramped vessels over 6 h to targeted salinities via water delivered from head tanks using peristaltic pumps. Head tanks contained holding tank water brought to target salinities using Instant Ocean, and we conducted four replicates for each of the five target salinities. We checked and removed mortalities and recorded water quality hourly during the ramping phase, followed by monitoring at each designated time point (0–6, 12, 24, 30 and 48 h). After 48 h, we removed containers individually, euthanized and counted fish to confirm survival. We conducted juvenile and adult AS_max_ experiments by allowing fish to remain in recirculating tanks while Instant Ocean was added to sumps over a 6 h ramp to targeted salinities. Three salinities were chosen [2.3 (control), 18.5 and 34.0 ppt] based on chronic salinity experiment results, preliminary acute experiments demonstrating these stages to be resistant to lower salinities (L. M. Komoroske, unpublished data), and because these ontogenetic stages are more likely to experience higher salinities *in situ* (Bennett, 2005). We monitored tanks hourly for mortalities and water quality parameters during the ramping phase, at each designated time point (0–6, 12, 24 and 48 h) and daily up to 3 weeks. At the termination of the adult AS_max_ experiment, fish were randomly selected from each of the three salinity treatments for CT_max_ trials to assess differences in thermal tolerance due to sublethal salinity stress.

### Statistical analyses

We performed all statistical analyses using R (version 2.15.2; R-CoreTeam, 2012) and associated packages ‘lme4’, ‘car’ and ‘multcomp’ (Hothorn *et al.*, 2008). We analysed data using linear models (LMs) via the R core package (R-CoreTeam, 2012) and generalized linear mixed models (GLMMs) using ‘lme4’ (Bates *et al.*, 2011). We generated model summary tables using ‘car’ (Fox and Weisberg, 2011) and conducted multiple comparisons for both LMs and GLMMs between treatment levels of fixed effects using ‘multcomp’ (Hothorn *et al.*, 2008). For CLT_max_ analysis, we employed GLMMs with a binomial error distribution and logit link function (Bates *et al.*, 2011) to determine differences in CLT_max_ among stages, including a random individual fish effect to account for repeated observations. We evaluated overdispersion by estimating the ratio of residual deviance to residual degrees of freedom (Dobson, 2002). To generate model estimates and confidence intervals for the fixed effects, we sampled from a naïve posterior distribution (60 000 times) for each stage of chronic temperatures at which 50% (CLT_max50_) and 95% (CLT_max95_) mortality would occur (McElreath, 2013). For CT_max_ analysis, we conducted two separate LMs because larval and late-larval fish were available at only one acclimation temperature. We applied the first LM to all ontogenetic stages at the medium acclimation temperature only, using ontogenetic stage as a single predictor of LOE. The second LM for post-larval fish employed ontogenetic stage (juvenile, adult and post-spawning adult), acclimation temperature and their interaction as predictors of LOE (R-CoreTeam, 2012). Fish size within each ontogenetic stage did not significantly affect CT_max_ for any acclimation group and was therefore not included in the final LMs as a covariate. We evaluated data assumptions and LM fit graphically, i.e. residual vs. fitted values, residual vs. predictor values and residual histograms (Zuur *et al.*, 2009).

We assessed effects of salinity and exposure duration for AS_max_ with the same approach described for CLT_max_, using separate logit link GLMMs for: (i) the late-larval stage and (ii) juveniles and adults. We also followed the juvenile–adult model with a GLMM to evaluate differences between ontogenetic stage and salinity specifically after 96 h of exposure (logit link function, including random effect of tank). Finally, we evaluated the effect of salinity on CT_max_ in adult fish following AS_max_ exposures using an LM with salinity as a single predictor of LOE. Pairwise comparisons were performed for all analyses using the glht() function in the ‘multcomp’ package, with an adjusted α = 0.05.

## Results

### Thermal tolerance

**Figure 2:**
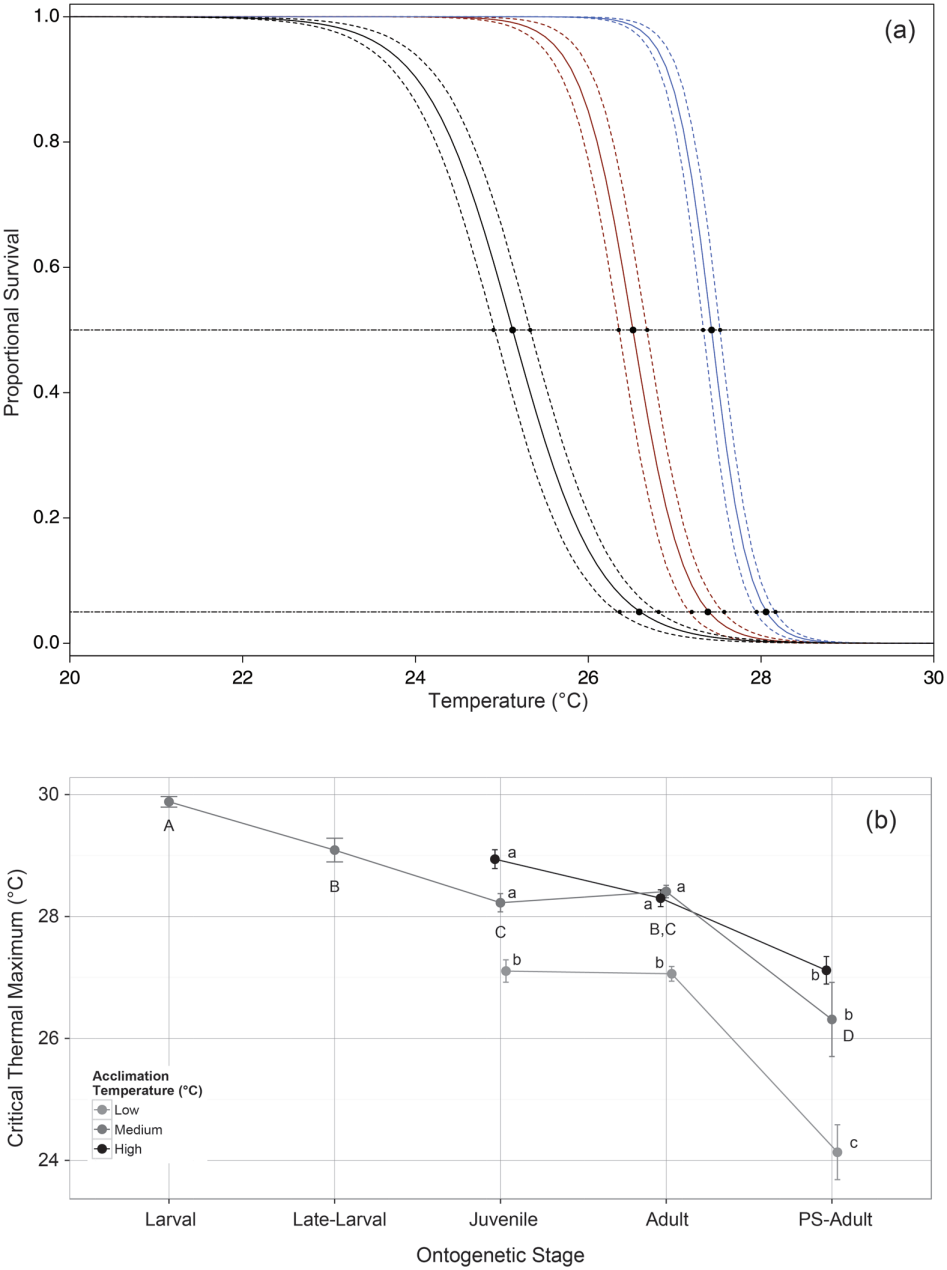
Thermal tolerance of Delta Smelt. (**a**) Estimates of chronic lethal thermal maximum (CLT_max_; continuous lines) and 95% confidence intervals (dashed lines) for juvenile (blue), adult (red) and post-spawning Delta Smelt (black). Points denote the CLT_max50_ and CLT_max95_ estimated mean and 95% confidence interval for each ontogenetic stage on the respective curves. (**b**) Critical thermal maximum (CT_max_) temperatures (means ± SEM) for ontogenetic stages of Delta Smelt at different acclimation temperatures, with jitter (0.05) added to each point to avoid overlapping. Capital letters denote significantly different groups across all ontogenetic stages at medium acclimation temperature only; lower case letters denote significantly different groups among stages of non-larval fish at low, medium and high acclimation temperatures. Within each case, groups not sharing a common letter are significantly different at an adjusted α level of 0.05 as determined by a linear model and pairwise comparisons. PS-Adult denotes post-spawning Delta Smelt.

Delta Smelt exhibited decreasing thermal tolerance across successive ontogenetic stages over both chronic and acute time scales. In chronic exposures, CLT_max_ for post-larval stages revealed decreasing upper thermal acclimation limits across successive ontogenetic stages (Fig. 2a and Table 1; *post hoc* adjusted *P* < 0.001). Temperature estimates for both 50% mortality (CLT_50_) and 95% mortality (CLT_95_) were below CT_max_ for each ontogenetic stage (Table S2).

**Table 1:** Model results for Delta Smelt thermal tolerance

CT_max, medium acclimation all stages_	SS	Df	F	*P*-value
Ontogenetic stage	81.45	4	29.82	<0.001*
Residuals	53.95	79		

CT_max, post-larval stages_	SS	Df	F	*P*-value
Ontogenetic stage	151.43	2	92.22	<0.001*
Acclimation temperature	117.09	2	71.31	<0.001*
Ontogenetic × acclimation temperature	15.24	4	4.64	0.001*
Residuals	171.57	209		

CLT_post-larval stages_	Wald χ^2^	Df	*P*-value	
Temperature	2300.39	1	<0.001*	
Ontogenetic stage	263.47	2	<0.001*	
Temperature × ontogenetic stage	262.52	2	<0.001*	

The CT_max_ analyses used linear models (Gaussian error distributions), and CLT analysis employed a generalized linear mixed model (binomial error distribution). See text for pairwise comparisons.

Acute thermal tolerance of Delta Smelt (CT_max_) across all stages examined at the medium acclimation temperature was highest in larval fish (Fig. 2b, Table 1 and Table S2). The CT_max_ decreased with each subsequent ontogenetic stage (adjusted *P* ≤ 0.05), with the exceptions of between adults and juveniles (adjusted *P* = 0.95) and between adults and late-larval fish (adjusted *P* = 0.087).

For post-larval stages of Delta Smelt, acclimation temperature, ontogenetic stage and their interaction were significant factors influencing CT_max_ (Fig. 2b, Table 1 and Table S2). Within each stage, CT_max_ for the lowest acclimation group was significantly reduced relative to both medium and high acclimation temperature groups (adjusted *P* ≤ 0.03; Fig. 2b and Tables S2 and S3). However, the CT_max_ values of medium and high acclimation temperature groups did not differ from each other (adjusted *P* ≥ 0.24), indicating that effects of acclimation on thermal tolerance are minimal at higher temperatures in Delta Smelt.

### Warming tolerance

**Figure 3:**
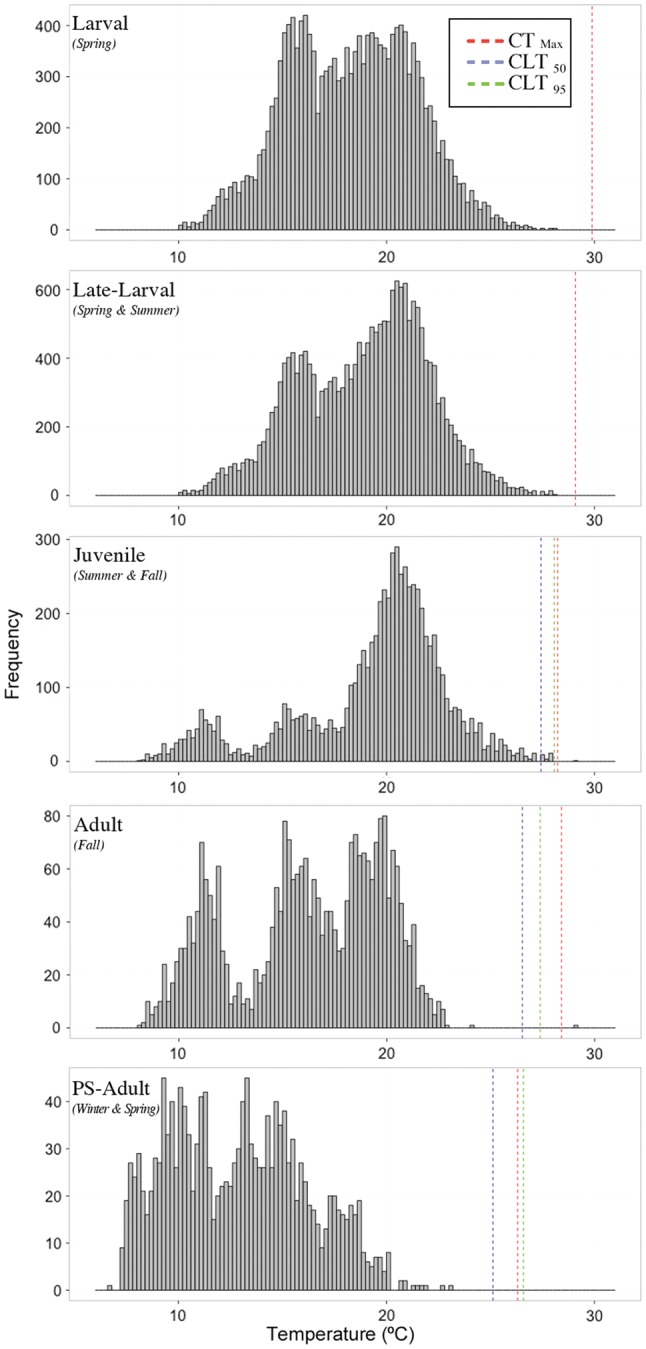
Habitat temperature profiles for each ontogenetic stage of Delta Smelt examined and the corresponding thermal tolerances determined in this study. The histograms depict constructed data sets of water temperature distributions relevant for each ontogenetic stage; red dashed lines indicate the mean CT_max_ for each stage of fish at medium acclimation temperature; dashed blue and green lines depict the CLT_max50_ and CLT_max95_, respectively, for each stage in post-larval fish.

Using current median environmental temperature metrics for *T*_habitat_, all Delta Smelt ontogenetic stages exhibited a WT >8°C, with juvenile Delta Smelt exhibiting the lowest warming tolerance followed by late-larval fish (Fig. 3 and Table 2). However, applying additional *T*_habitat_ metrics that encompass extreme events with potential important biological consequences, the WT of all stages was greatly reduced (Table 2). In rare events, observed habitat temperatures exceeded the CT_max_ for juvenile and adult Delta Smelt, resulting in WT <0. Although post-spawning Delta Smelt have lower thermal tolerance relative to other stages, they had the highest WT due to expected seasonal timing of their presence (January–May). However, these results do not evaluate WT of fish surviving first-year spawning throughout subsequent seasons, potentially to spawn a second year, because no adequate thermal tolerance data were available for Delta Smelt beyond the first-year post-spawning stage.

**Table 2:** Warming tolerance estimates for each stage of Delta Smelt, defined as $\overline{\hbox{CT}_{{\rm max}}}{}_{\rm (medium acclimation temperature)}$ − *T*_habitat (metrics of habitat temperatures)_

Ontogenetic stage	*T*_habitat_			
	Median	95th _centile_	99th _centile_	Maximum
30 dph	11.58	6.88	4.98	1.88
60 dph	9.79	5.49	3.59	1.09
Juvenile	8.03	4.13	2.03	−0.77
Adults	11.91	7.41	6.31	−0.59
PS-Adults	13.41	7.81	6.31	3.21

### Salinity tolerance

**Figure 4:**
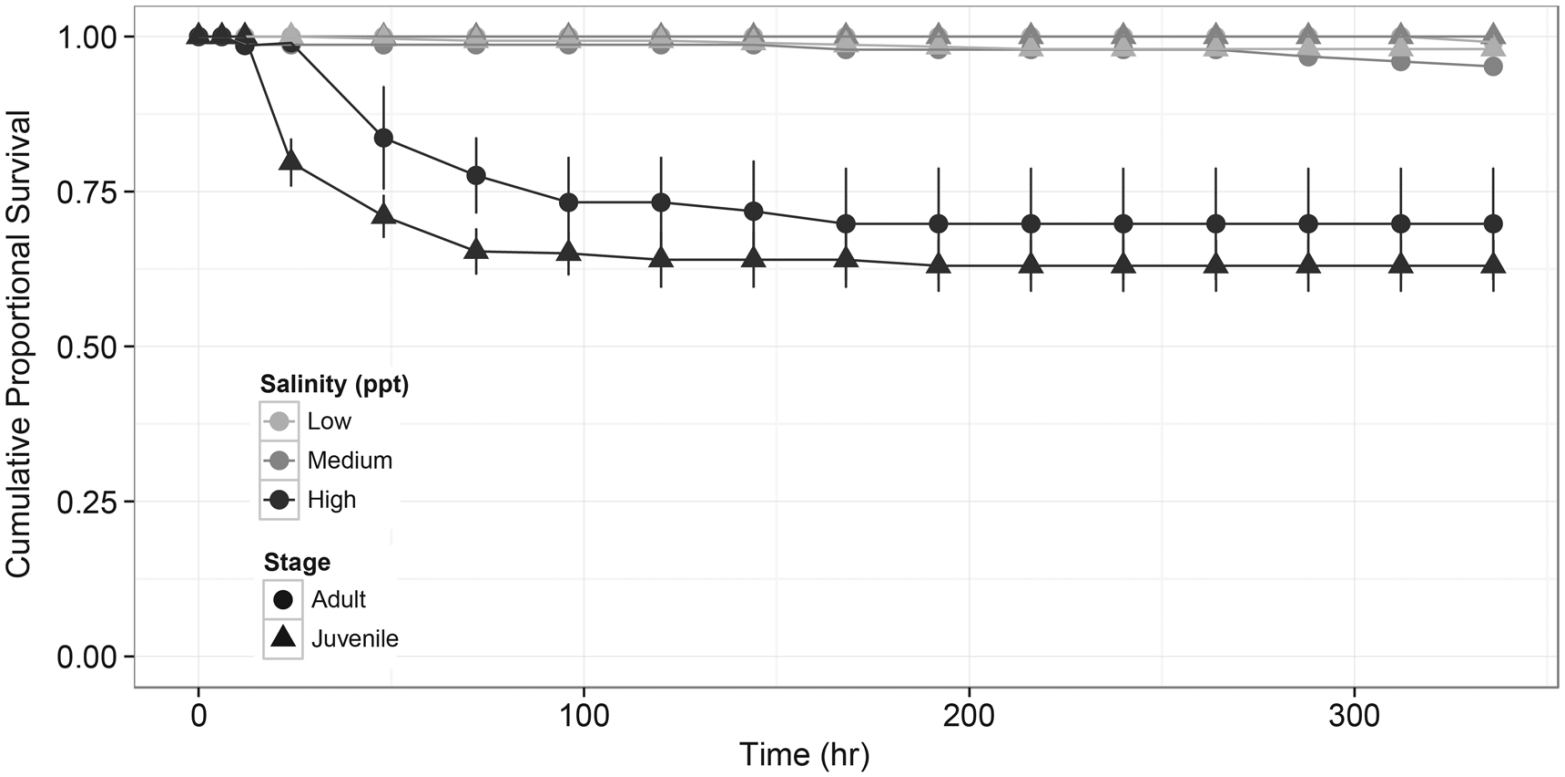
Cumulative proportional survival of juvenile and adult Delta Smelt in acute salinity maximum over 2 weeks of exposure (low salinity, 2.3 ppt, medium salinity, 18.5 ppt; and high salinity, 34.0 ppt). Late-larval data are not shown because they did not exhibit any significant difference in survival across salinity treatments.

Increased salinity affected Delta Smelt survival only in extreme conditions, and was dissimilar among stages. In AS_max_ exposures, salinity did not affect survival of late-larval fish among any environmentally relevant salinities (0.4–18 ppt; Table 3); however, both juvenile and adult survival was negatively affected by increased salinity (Fig. 4 and Table 3). For adults and juveniles, principal mortality occurred between 24 and 96 h; after 96 h, survival was reduced in the highest treatment (34.0 ppt) relative to medium (18.5 ppt) and control (2.3 ppt), and survival of juveniles was significantly lower than that of adults (*post hoc* adjusted *P* < 0.001). Survival in AS_max_ experiments at 96 h and 2.3 ppt was 100% for adults and 99.4% for juveniles; at 18.5 ppt it was 99.2% for adults and 100% for juveniles; and at 34.0 ppt it was 81.5% for adults and 64.5% for juveniles. These results indicate that a substantial proportion of Delta Smelt in these stages can withstand even extreme changes in salinity conditions. Furthermore, in CLS_max_ experiments, salinity did not affect adult or juvenile survival [survival was 100% for adults (*n* = 153) and 99.0% for juveniles (*n* = 287)], which covered the same salinity range (2.3–34.0 ppt), but with a slower rate of increase (2 ppt/12 h). We also did not detect delayed mortality in fish held at 34 ppt for 3 weeks after CLS_max_ exposures (survival at 3 weeks was  99.3% for adults and 99.0% for juveniles). Finally, CT_max_ among surviving adult Delta Smelt at the termination (14 days) of the AS_max_ experiments did not differ among 2.3, 18.5 and 34.0 ppt treatments (Table 3), indicating that acute salinity exposure did not impact thermal tolerance.

**Table 3:** Model results for Delta Smelt salinity tolerance

Acute salinity_juvenile and adult over 14-day exposure_	Wald χ^2^	Df	*P*-value	
Salinity	24.78	3	<0.001*	
Ontogenetic stage	0.424	2	0.809	
Experimental hour	407.0	1	<0.001*	
Salinity × stage	7.742	2	0.021*	
Salinity × experimental hour	2.775	2	0.250	
Stage × experimental hour	0.075	1	0.784	
Salinity × stage × experimental hour	0.004	2	>0.998	

Acute salinity_juvenile and adult at 96 h exposure_	Wald χ^2^	Df	*P*-value	
Salinity	66.2682	2	<0.001*	
Ontogenetic stage	10.2204	1	0.001*	
Salinity × stage	0.0002	2	>0.999	

Acute salinity_late-larval 48 h exposure_	Wald χ^2^	Df	*P*-value	
Salinity	0.183	4	0.996	
Experimental hour	9.803	6	0.133	
Salinity × experimental hour	6.413	24	0.999	

CT_max, adults post 2-week salinity exposure_	SS	Df	F	*P*-value
Salinity	1.574	2	1.61	0.209
Residuals	27.40	56		

We used generalized linear mixed models (binomial error distributions) to analyse acute salinity exposures, a linear model (Gaussian error distributions) CT_max_ across salinity groups. See text for pairwise comparisons.

## Discussion

Understanding how organisms will respond to climate change is critical if conservation and management strategies are to be successful in the long term (Helmuth, 2009; Hoffmann and Sgro, 2011). The physiological capacity of organisms to cope with predicted alterations in abiotic conditions is a critical component of their responses to climate change, and can be heavily influenced by phenotypic plasticity and life history (Stillman, 2003; Wikelski and Cooke, 2006; Pörtner and Farrell, 2008). The thermal tolerance of Delta Smelt generally decreased among successive ontogenetic stages, and they had limited capacity to increase tolerance via thermal acclimation. Juvenile Delta Smelt exhibited the lowest WT, and rare maximal temperatures *in situ* have already been observed that exceed tolerance limits of juvenile and adult Delta Smelt. In contrast, the salinity tolerance of Delta Smelt spanned the range of expected environmental conditions for each ontogenetic stage; however, salinity did impact juvenile and adult survival under high fluctuation.

The higher thermal tolerance we observed in larval Delta Smelt relative to older stages is consistent with their developmental and evolutionary history. Delta Smelt swim bladder and fin development are not complete until ∼65 dph (∼20 mm total length; Mager *et al.*, 2004), when they can fully control their buoyancy and efficiently use tidal and river currents to migrate. Before this time, they are likely to have limited control over their movements and are thought to be mostly demersal in shallow shoaling sandy areas (Mager *et al.*, 2004; Moyle *et al.* 2010) where temperatures can quickly increase. The understanding of Delta Smelt larval behaviour and habitat preferences is currently limited to a few laboratory and field survey studies (Baskerville-Bridges *et al.*, 2004; Dege and Brown, 2004; Mager *et al.*, 2004), and Delta Smelt larvae have been observed in deeper river channel habitats (Grimaldo *et al.*, 2004). However, if Delta Smelt have indeed evolved to use shallow shoaling sandy areas during these early ontogenetic stages, this could contribute to the increased thermal tolerance we observed in larval and late-larval stages. Organisms with limited mobility may exhibit higher environmental stress tolerance relative to mobile organisms that can cope behaviourally by moving to areas with favourable environmental conditions (Menge and Olson, 1990), so it may be that larval Delta Smelt with limited mobility may experience selective pressures favouring enhanced thermal tolerance. There is also some evidence that water export pumping schedules in the SFE may recently have favoured survival of smaller, late-spawned larval fish (Bennett *et al.*, 2008; Bennett, 2011). If this is the case, anthropogenically driven selection coupled with seasonal conditions could also contribute to enhanced thermal tolerance because late-spawned larval fish must be able to survive in warmer late spring and early summer shallow waters.

Species with the highest risk of extinction from climate change are those that have little tolerance for warming, limited acclimation capacity and tight constraints on dispersal (Deutsch *et al.*, 2008). While these concepts have primarily been taken to confer that tropical species are at higher risk relative to temperate species, these criteria are also met by many endemic aquatic species. These species include temperate fishes (Ficke *et al.*, 2007) and invertebrates (Muhlfeld *et al.*, 2011) that are adapted to regional conditions and confined to lakes, riverine or estuarine ecosystems that offer few avenues of dispersal. Native SFE fishes are adapted to the local abiotic conditions and have very little ability to disperse poleward if water temperatures make their current habitat unsuitable. As an endemic fish with largely non-overlapping generations (Bennett, 2005), Delta Smelt population persistence relies on individuals surviving high larval mortality pressures each year to reach reproductive stages. Coupled with water temperatures being closest to juvenile tolerance limits, these life-history dynamics potentially make Delta Smelt especially susceptible to population decline from a single hot year. Conversely, providing suitable thermal habitat for juveniles can also potentially have positive impacts on the population. Understanding these relationships provides insight into where and when to target management efforts. Recent climate-change assessments indicate that SFE waters are likely to become warmer and the low-salinity zone may move further upstream, limiting optimal habitat for Delta Smelt in the absence of mitigation actions (Brown *et al.*, 2013). Effective conservation strategies to ensure that Delta Smelt habitat maintains suitable thermal conditions during summer and autumn may prove to be critical for the sustainability of this species in the wild.

Post-spawning Delta Smelt had higher WT despite their lowered tolerances because of the seasonal timing of this stage during winter and spring. However, a small percentage of adults are estimated to survive their first year post-spawning (Bennett, 2005), but to reach a second reproductive season they must be able to cope with environmental fluctuations throughout the following year. Second-year fish have higher fecundity (Bennett, 2005) but are rarely observed *in situ*, limiting their reproductive contribution to the population. While the lack of 2-year-old Delta Smelt in the wild may be attributable to many factors, such as food limitation, disease or susceptibility to predation, if they are not able to improve their tolerance after recovering from spawning, the thermal sensitivity of post-spawning fish may also play an important role in limiting their presence due to heightened water temperatures in summer and early autumn. The substantially lowered thermal tolerance of post-spawning adults also underscores the importance of considering timing of abiotic stressors with biological stressors, such as the energetic costs of gonadal development, migration and spawning, in evaluating species' sensitivity to climate change (Perry *et al.*, 2005; Pörtner and Farrell, 2008).

In complex ecosystems with multiple stressors, such as the SFE, isolation and evaluation of the effects of individual factors is critical to understanding their contribution to observations *in situ*. Despite the high salinity tolerance we observed, the distribution of juvenile and adult Delta Smelt in the SFE has been strongly correlated with the low-salinity zone (1–6 ppt; Bennett, 2005; Feyrer *et al.*, 2011). This suggests that other factors limit their ability to expand into high salinities, such as reduced physiological performance due to osmoregulatory costs (Hasenbein *et al.*, 2013) or other ecological elements that co-vary with salinity, e.g. marine predators, food resources or habitat structure (Bennett, 2005). Thus, while the fundamental niche of Delta Smelt encompasses a wide salinity range, their realized niche may be principally in the low-salinity zone (Hutchinson, 1957), and the combined impacts of climate change and increased anthropogenic resource demands pushing the low-salinity zone further upstream could reduce Delta Smelt optimal habitat.

Our study demonstrates the need to consider life history in assessing climate-change impacts, particularly for species adapted to spatially and temporally heterogeneous environments. Synergistic effects between climate and other anthropogenic threats have been predicted to intensify climate-change impacts in other systems (Harley *et al.*, 2006), and are also likely to occur in the highly anthropogenically modified SFE unless effective conservation approaches are implemented. With the multitude of biological stressors and competing human resource use needs in the SFE, this will undoubtedly be very challenging; however, understanding the physiological capacity of sensitive organisms to cope with altered temperature and salinity regimens is critical to the development of successful conservation and restoration strategies.

## Supplementary material

Supplementary material is available at *Conservation Physiology* online.

## Funding

This work was supported by the University of California Agricultural Experiment Station [grant number 2098-H to N.A.F.], the United States Department of Interior, Bureau of Reclamation [contract number R12AP20018 to R.E.C. and N.A.F.], the State and Federal Contractors Water Agency [grant number 201301957 to R.E.C.] and the California Delta Stewardship Council [contract number 201015533 to R.E.C. and N.A.F.]. Partial student funding was provided to M.H. by the Bavarian Elite Programme Universität Bayern e.V.-Scholarship for graduate students and post-graduate students, and to L.M.K. by the National Science Foundation Graduate-12 Fellowship Program [under DGE grant number 0841297 to S. L. Williams and B. Ludaescher] and the California Sea Grant Delta Science Doctoral Fellowship R/SF-56.

## Supplementary Material

Supplementary Data
