# Supplementary material for: Ontogeny influences sensitivity to climate change stressors in an endangered fish
Source: Conserv Physiol. 2014 Mar 10;2(1):cou008. doi: 10.1093/conphys/cou008 (PMC4806739; doi:10.1093/conphys/cou008)
Supplement: Supplementary Data [file supp_2_1_cou008__index.html]

Ontogeny influences sensitivity to climate change stressors in an endangered fish — Supplementary Data 

# Ontogeny influences sensitivity to climate change stressors in an endangered fish

## Supplementary Data

Supplementary Data

**Files in this Data Supplement:**

- Supplementary Data - Docx file
