# Supplementary material for: Ontogeny influences sensitivity to climate change stressors in an endangered fish
Source: Conserv Physiol. 2014 Mar 10;2(1):cou008. doi: 10.1093/conphys/cou008 (PMC4806739; doi:10.1093/conphys/cou008)
Supplement: Supplementary Data [file supp_cou008_cou008supp.docx]

**Appendix 1: Supplementary Tables**

Table S1: (a) Temperature acclimation summaries (mean ± SD) of three week acclimation period (b) Fish weights (weighed as wet mass ± 0.1g) and lengths (measured as fork length ± 1.0 mm) for post-larval delta smelt used in experiments (mean ± SD).

*(a) Temperature Acclimation Group*

Stage High Medium Low

Larval 16.4 ± 0.3

Late-larval 16.4 ± 0.3

Juvenile 19.7 ± 0.2 15.7 ± 0.1 11.9 ± 0.1

Adult 18.7 ± 0.2 16.6 ± 0.1 12.0 ± 0.2

PS-Adult 18.7 ± 0.2 15.3 ± 0.1 12.4 ± 0.1

*(b)*

*Fish length (mm) Fish weight (g)*

Juvenile 49 ± 8 0.8 ± 0.4

Adult 56 ± 7 1.3 ± 0.5

PS-Adult 70 ± 8 3.9 ± 1.4

Table S2. Basic statistics of delta smelt thermal tolerance: (a) CT_Max_ of delta smelt by ontogenetic stage and acclimation temperatures (b) CLT_Max_ 50% and 95% predictions.

*(a) CT_Max_*

*Accl. Temp. Stage N Mean SD 95% C.I.*

Low Juvenile 20 27.1 0.8 26.7-27.5

Adult 20 27.1 0.5 26.8-27.3

Adult-PS 18 24.1 1.9 23.2-25.1

Medium larval 16 29.9 0.3 29.7-30.1

late-larval 19 29.1 0.9 28.7-29.5

Juvenile 20 28.2 0.7 27.9-28.5

Adult 20 28.4 0.5 28.2-28.6

Adult-PS 9 26.3 1.8 24.9-27.7

High Juvenile 20 28.9 0.7 28.6-29.3

Adult 20 28.3 0.6 28.0-28.6

Adult-PS 12 27.1 0.8 26.6-27.6

*(b) CLT_Max_*

*Stage N Estimate 95% C.I.*

Juvenile 331 27.4 27.3-27.5

CLT_50%_ Adult 231 26.5 26.4-26.7

PS-Adult 349 25.1 24.9-25.3

Juvenile 331 28.1 28.0-28.2

CLT_95%_ Adult 231 27.4 27.2-27.6

PS-Adult 349 26.6 26.4-26.8

Table S3. Linear regressions for acclimation temperature effects on CT_max_ for post-larval stages of delta smelt. In each model, m = slope and b = y-intercept of the model.

*Stage Formula (y = mx + b)*

Juvenile CT_max_ = 0.245 accl. temp + 24.2

Adult CT_max_ = 0.209 accl. temp + 24.7

Adult-PS CT_max_ = 0.508 accl. temp + 17.9
